# Metalloproteins as risk factors for osteoarthritis: improving and understanding causal estimates using Mendelian randomization

**DOI:** 10.1007/s10067-024-06968-7

**Published:** 2024-05-08

**Authors:** Jiaze Li, Mingyang Guan, Lin Qi, Fengping Zhang, Chenxu Jia, Qingtao Meng, Jian Han

**Affiliations:** Department of Orthopedics, Dalian Third People’s Hospital Affiliated to Dalian Medical University, Dalian City Third People’s Hospital, Dalian, 116091 Liaoning Province China

**Keywords:** Genetic analyses, Mendelian randomization, Metalloproteins, Osteoarthritis

## Abstract

Osteoarthritis (OA) is one of the most prevalent musculoskeletal disorders and a primary cause of pain and disability among the elderly population. Research on the relationship between metalloproteins (MPs) and OA is limited, and causality remains unclear. Our objective is to utilize Mendelian randomization (MR) to explore the possible causal relationship between MPs and OA. The data on MPs were derived from a Genome-Wide Association Study (GWAS) analysis involving 3301 samples. The GWAS data for OA were obtained from an analysis involving 462,933 European individuals. In this study, a variety of two-sample Mendelian randomization methods (two-sample MR) to evaluate the causal effect of MPs on OA, including inverse variance weighted method (IVW), MR-Egger method, weighted median method (WM), simple mode, weight mode, and Wald ratio. The primary MR analysis using the IVW method reveals a significant negative correlation between Metallothionein-1F (MT-1F), zinc finger protein 134 (ZNF134), calcium/calmodulin-dependent protein kinase type 1D (CAMK1D), and EF-hand calcium-binding domain-containing protein 14 (EFCAB14) with the occurrence of osteoarthritis (OA) (*p* value < 0.05). However, no causal relationship was observed in the opposite direction between these MPs and OA. Notably, even in combined models accounting for confounding factors, the negative association between these four MPs and OA remained significant. Sensitivity analysis demonstrated no evidence of horizontal pleiotropy or heterogeneity, and leave-one-out analysis confirmed the robustness of the results. In this study, we have established a conspicuous association between four distinct MPs and OA. This discovery augments our understanding of potential avenues for the diagnosis and treatment of this condition.

**Table:** 

**Key Points** • *The MR method was employed to assess the relationship between MPs and OA.* • *A total of four types of MPs have demonstrated inhibitory effects on the occurrence of OA.*

**Supplementary Information:**

The online version contains supplementary material available at 10.1007/s10067-024-06968-7.

## Introduction

Osteoarthritis (OA) is the most prevalent degenerative joint disease, affecting over 303.1 million people worldwide [1]. Due to rising obesity rates and an aging population, its incidence is on the rise, becoming a major cause of disability [2]. In addition to its impact on physical function, OA also has negative effects on mental health. There is increasing evidence suggesting that patients with knee or hip OA are more likely to experience depression and even have higher rates of suicidal ideation [3]. Currently, apart from total joint replacement surgery, advanced-stage OA lacks other effective treatment modalities [4]. Therefore, it is necessary to investigate the etiology of OA in order to prevent the development of the condition.

Metalloproteins (MPs) are a generic term for a protein that contains a metal ion cofactor [5]. Within living organisms, MPs are widely distributed, encompassing multiple categories [6]. They not only catalyze a variety of vital biochemical reactions but also play crucial roles in maintaining metal ion metabolism and homeostasis within the organism [7]. In recent years, the association between certain MPs and OA has garnered considerable attention. Studies have revealed that in OA patients, the expression of metallothionein-1 (MT-1) is upregulated [8]. MT-1 has the ability to suppress the expression of pro-inflammatory cytokines in synovial cells of OA patients, thereby slowing the progression of OA [9]. However, a comprehensive investigation into the relationship between MPs and OA is still relatively limited. Consequently, further research is needed to validate the causal link between MPs and OA, providing theoretical support for future studies and clinical treatments.

Mendelian randomization (MR) is a genetic variable analysis method that follows Mendel’s laws of inheritance [10]. It uses genetic variation as IVs to infer whether a risk factor causally influences health outcomes. MR is particularly valuable in certain situations, especially when conducting randomized controlled trials for causal relationships is challenging or when observational studies produce biased associations due to confounding or reverse causality [11]. MR addresses these issues by utilizing genetic variations that are associated with the exposure factor as IVs, as the alleles of these genetic variations are randomly assigned and not influenced by reverse causality. It is worth noting that in the past decade, many candidate gene and genome-wide association studies (GWAS) have been published, enabling MR studies to leverage these reported associations without the need for recruiting new patients or conducting additional study designs [12].

This study employed a large-scale GWAS dataset and conducted a two-sample MR investigation to analyze MPs markers potentially causally linked to OA. This research may contribute to unraveling the genetic features and biological mechanisms underlying osteoarthritis.

## Materials and methods

### Research report guidelines and study design

**Fig. 1:**
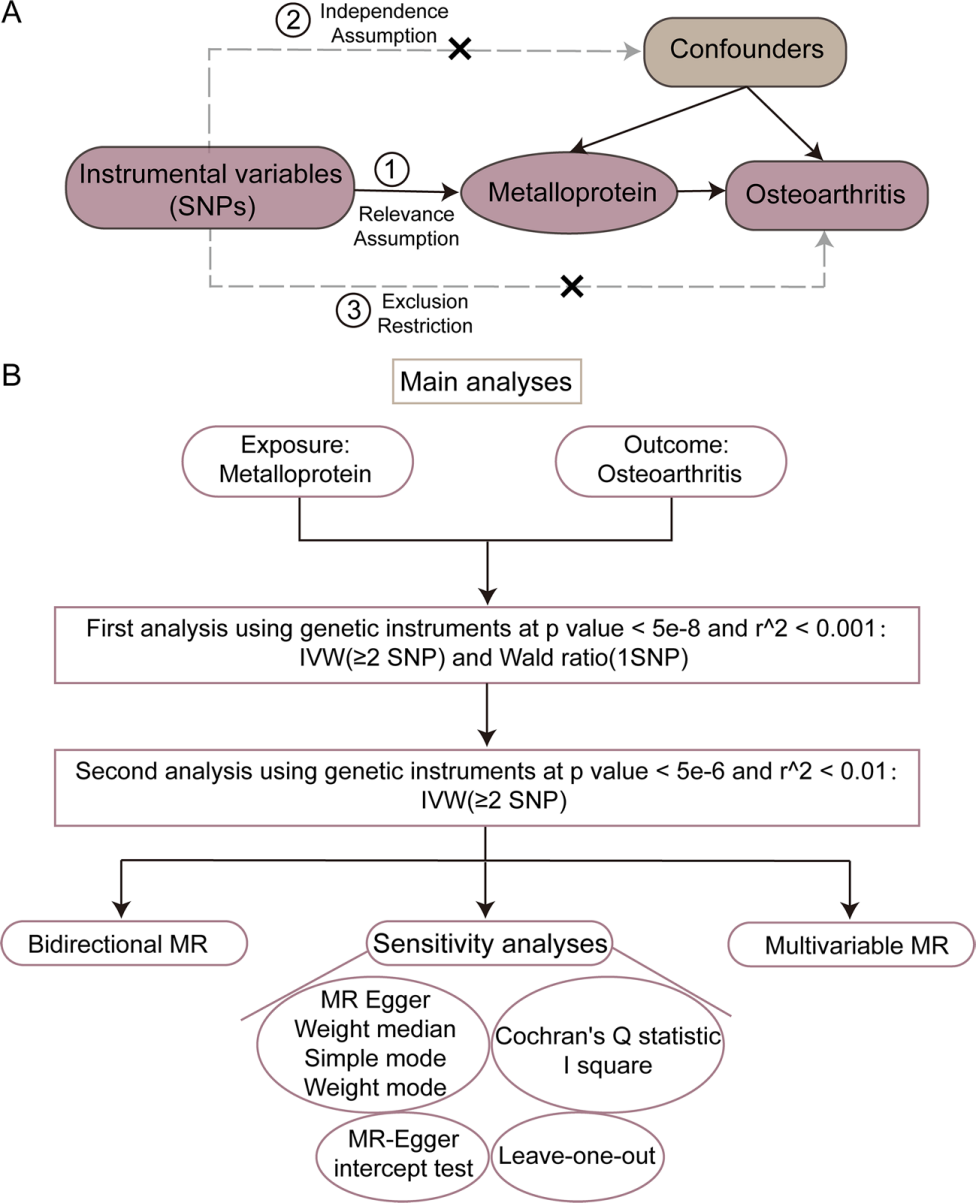
Flow chart for the two-sample Mendelian randomization analysis of OA. **A** Schematic diagram of the basic assumptions of MR analysis. The basic assumptions include (1) relevance assumption, that is, the selected IVs must be significantly correlated with exposure factors; (2) independence assumption, that is, IVs must have no significant correlation with potential confounding factors that might affect exposure or outcomes; and (3) exclusion restriction, that is, IVs could only affect the outcome through the path of “IVs → exposure → outcome,” and IVs cannot directly affect the outcome. **B** General flowchart of the analytical method of this study. SNP, single nucleotide polymorphism; IVW, inverse variance weighted; MR, Mendelian randomization; IVs, instrumental variables

This study employed a two-sample MR investigation along with publicly available datasets to investigate the causal relationship between MPs and the risk of OA. The research report is based on the Mendelian randomization reporting guidelines Strengthening the Reporting of Observational Studies in Epidemiology Using Mendelian Randomization (The STROBE-MR Statement) [13].A schematic diagram illustrating the research design is presented in Fig. 1.

### Data source

We identified five distinct categories of MPs as our primary exposure factors, encompassing zinc finger proteins (ZNF), ferritin-related proteins (FRP), calcium-related proteins (CRP), matrix metalloproteinases (MMPs), and metallothioneins (MTs). These categories collectively comprise a total of 65 unique proteins. OA was chosen as the target outcome for investigation. The GWAS datasets corresponding to these five MPs categories were extracted from the comprehensive GWAS analysis of 3301 individuals of European ancestry conducted by Benyamin et al. [14], constituting a dataset encompassing 3301 individual samples. Specific details pertaining to these datasets are comprehensively outlined in Table S1. Furthermore, the GWAS data for OA were obtained from the extensive analysis conducted by Ben Elsworth et al., comprising a cohort of 462,933 individuals of European descent. This dataset encompasses 38,472 OA samples and 424,461 control samples. The primary data source emanates from the UK Biobank (https://www.ukbiobank.ac.uk/), and additional particulars can be referenced in Table S2.

### Instrumental variable selection and strength evaluation

Genetic variants can be considered instrumental variables (IVs) only when they satisfy the three stringent assumptions outlined below [15]: First and foremost, the relevance assumption demands that the selected independent variables (IVs) display a significant association with the factors influencing exposure. Secondly, following the Independence Assumption, it is imperative that IVs do not exhibit any notable correlations with potential confounding factors capable of influencing both the exposure and the resulting outcome. Lastly, the Exclusion Assumption asserts that IVs can exclusively influence the outcome through the established causal pathway “IVs → exposure → outcome” and must refrain from exerting any direct influence on the outcome itself.

In this study, single nucleotide polymorphisms (SNPs) were employed as IVs. The initial screening criteria for SNP analysis within the five major categories of MPs were as follows: SNPs associated with MPs in GWAS with a *p* value < 5e-8 were considered the primary screening condition, with the simultaneous exclusion of SNPs in linkage disequilibrium (LD). Further criteria included SNPs with an *r*^2^ < 0.001 and a minimum physical distance of 10,000 kb between each pair of genes.

For secondary analysis, the screening criteria were as follows: A *p* value < 5e-6 was employed as the primary screening condition, with the exclusion of LD SNPs having an *r*^2^ < 0.01 and a minimum physical distance of 10,000 kb between genes. Subsequently, the identified SNPs were cross-referenced with the GWAS data for OA and harmonized. Additionally, the F-statistic was computed to assess the bias of weak IVs [16]. When F < 10, it signifies that the genetic variation used serves as a weak IVs, potentially introducing bias into the results, and thus, such variables were excluded to prevent any impact on the outcomes.

### Estimation of MR causal effect

In this study, we employed various two-sample MR methods to evaluate the causal effect of MPs on OA [17–21]. These methods included the inverse variance weighted method (IVW), the MR-Egger method, the weighted median method (WM), the simple mode, the weighted mode, and the coefficient ratio method (Wald ratio). The distinctive feature of the IVW lies in its omission of the intercept term during regression, using the reciprocal of the outcome variance as the fitting weight. Thus, in the absence of pleiotropic effects, regardless of heterogeneity, and when the number of SNPs is greater than or equal to 2, the IVW method serves as the primary MR analysis approach in this study. Conversely, the Wald ratio employs a single IV to estimate the causal relationship between the exposure factor and the outcome when the number of SNPs is 1. In such cases, the Wald ratio is the primary MR analysis method, while the other four methods are used as supplementary tools. The screening criteria for both the IVW and the Wald ratio are set at a *p* value threshold of < 0.05.

### Sensitivity analysis

In this study, we employed various methods, including heterogeneity tests, pleiotropic tests, and leave-one-out tests, to assess the sensitivity of the analysis results, as outlined below.

#### Heterogeneity test

The Cochran Q test was used to evaluate the heterogeneity among the estimated values of each SNP [21]. If the Cochran Q test was statistically significant, it proved that the analysis results had significant heterogeneity. Because the Cochran Q test could only test the presence or absence of heterogeneity, it cannot test the distribution of heterogeneity. Therefore, use the *I* ^2^ statistic to reflect the proportion of the heterogeneous part of the IVs in the total variation: *I* ^2^ ≤ 0, set it to 0, indicating that no heterogeneity was observed; 0 < *I* ^2^ ≤ 0.25, suggesting mild heterogeneity; 0 0.25 < *I* ^2^. When ≤ 50%, it indicates moderate heterogeneity; when I ^2^ ≥ 50%, it indicates high heterogeneity.

#### Pleiotropic effect test

Pleiotropic effect assessment was conducted using the MR-Egger Intercept Testto examine the presence of pleiotropy in the IVs. The *p* value less than 0.05 in the MR-Egger Intercept Test indicates significant horizontal pleiotropy within the genetic variation. The fundamental assumption of MR for causal inference is the absence of horizontal pleiotropy, denoted by a *p* value greater than 0.05.

#### Leave-one-out test

This procedure systematically involves the exclusion of individual SNPs, one at a time, followed by the computation of MR results for the remaining IVs. The objective was to assess whether the presence or absence of a specific SNP significantly influenced the association between MPs and OA. If there was a substantial disparity between the MR effect estimation and the overall effect estimation following the removal of an IV, it signified that the MR effect estimation was highly sensitive to the presence or absence of that particular SNP.

### Bidirectional Mendelian randomization analysis

In our quest to discern the bidirectional causal relationship between OA and MPs, and to explore potential mutual causality, we conducted a comprehensive bidirectional Mendelian randomization analysis (bidirectional MR). In this study, we designated OA as the exposure factor and MPs as the outcome variable. Following meticulous matching and harmonization with the MPs dataset, we curated a set of SNPs that met stringent criteria to serve as reliable IVs.

#### SNP count ≥ 2

For this scenario, we applied the robust IVW as our primary MR analysis technique. This method facilitated the estimation of causal effects between OA and MPs, while concurrently mitigating potential biases.

#### SNP count = 1

In instances where only a single SNP was available, we relied on the Wald ratio as our primary MR analytical method.

To bolster the reliability of our analysis, supplementary methods were integrated, encompassing the MR-Egger method, the weighted median method, the simple mode, and the weighted mode. The assessment of causal effects revolved around the B (Beta) value as the primary evaluation index. We established a stringent screening criterion, mandating statistical significance at a *p* value threshold of < 0.05.

### Multivariate Mendelian randomization analysis

Multivariable Mendelian randomization (MVMR) is an extension of MR, which uses genetic variation associated with multiple potentially relevant exposures to estimate the impact of multiple exposures on a single outcome and could assess the impact of a single exposure factor on direct effects of outcome effects. We introduced nutritional anemias (Nutritional anemias, id: fin nb-D3_NUTRIANAEMIA), semilunar cartilage resection (Endoscopic resection of semilunar cartilage NEC, id: ukb-b-7881), malnutrition (Malnutrition, id: finn-b-E4_MALNUTRITION), and excessive and frequent menstruation with regular cycle (id: ukb-b-11572) 4 exposure factors, combined with MPs for MVMR analysis. We applied the screened MPs that has a significant causal association with OA, obtained common SNPs among multiple exposure factors through GWAS data, and matched and coordinated with OA harmonization, the obtained SNPs were used as IVs for MVMR analysis.

### Statistical analysis

All data processing and analysis in this article are based on R software (Version 4.2.2), mainly using R package TwoSampleMR [22] to carry out Mendelian randomization analysis, applying Cochran Q test and leave-one-out test. The robustness and reliability of the results were evaluated by the analytical method, and the genetic pleiotropic test was carried out by using the MR-Egger intercept test. When OA was used as the outcome, the evaluation indicators were OR and 95% CI, and when MPs was used as the outcome, the evaluation index is B (Beta) value. All statistical *p* value are two-sided tests, and the MPs related SNP sites generated by GWAS research in the first analysis are represented by *p* value < 5e-8 is considered statistically significant, and the MPs related SNP sites generated by GWAS research in the secondary analysis are represented by *p* value < 5e -6 that is considered statistically significant. Other statistical tests with *p* value < 0.05 is considered statistically significant.

## Results

### The first analysis of MPs on the risk assessment of OA

In this study, five MPs classes were used, including ZNF, FRP, CRP, MMPs, and MTs as the exposure factor and OA as the outcome variable. In order to get the IVs SNP, first use the parameter *p* value < 5e-8 Screen SNPs related to MPs, and remove SNP sites in linkage disequilibrium (*r* ^2^ < 0.001, kb = 1000); the obtained SNP sites were matched with the GWAS data ukb-b-14486 of OA, and after data harmonization, a total of 67 MPs were obtained IVs SNP; the remaining 26 MPs were not included in the first analysis, and the F statistics of all SNPs were greater than 10, indicating that most of the SNPs in the first analysis were IVs with strong effects and weak effects. The possible bias caused by IVs is limited (Table S3).

**Fig. 2:**
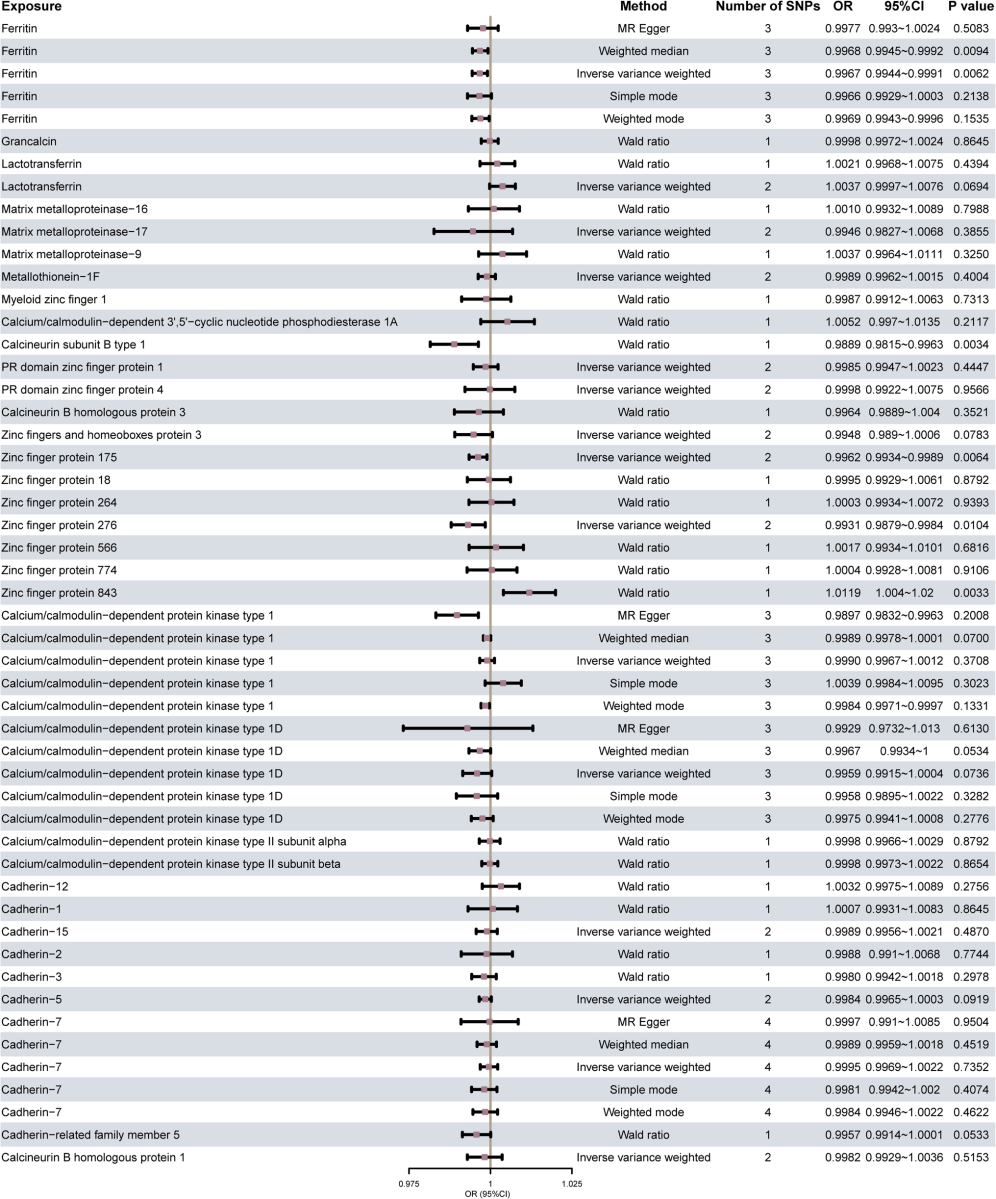
Estimation of causal effects between MPs and OA using different MR methods in first analysis. The forest plot shows the results of multiple MR model (method) analysis of the causal association between MPs (exposure) and OA, and the effect evaluation value is performed by OR (95% CI) display and at the same time display the number of SNPs in each model (number of SNPs) and *p* value (P value). MR, Mendelian randomization; SNP, single nucleotide polymorphism; OA, osteoarthritis; OR, Odds ratio; CI, confidence interval

Subsequently, the methods including IVW, MR-Egger method, weight median, simple mode, weight mode, and coefficient ratio were used respectively. A total of 6 kinds of MR including the Wald ratio method were used to estimate and analyze the causal effect, and the results are shown in Fig. 2. When the number of SNPs is ≥ 3, the IVW indicates 3 MPs: ferritin, ZNF276, ZNF175, and OA have a significant causal association (*p* value < 0.05); when the number of SNPs is 1, the Wald ratio indicates that 2 MPs: ZNF843, calcineurin subunit B type 1 and OA have a significant causal association (*p* value < 0.05).

Significant results were tested for heterogeneity using the Cochran Q test and I^2^ statistic (Table S4); the results show that ferritin, ZNF276, ZNF843, and calcineurin subunit B type 1 have no heterogeneity (*p* value > 0.05). At the same time, the pleiotropic effect test of the IVs SNP was conducted through the MR-Egger intercept test (Table S5); the results showed that the statistical hypothesis test *p* values of the intercept items of each index were greater than 0.05, and the intercept was close to 0, indicating that the causal inference of this study was not affected by horizontal pleiotropy.

### Secondary analysis of MPs on the risk assessment of OA

The stringent threshold initially employed limited our ability to analyze a broader range of intriguing MPs. In our pursuit of a deeper exploration into the causal relationship between MPs and the risk of OA, we adopted a more relaxed approach for SNP screening in a subsequent analysis. In this secondary analysis, we initiated the selection process by employing a parameter threshold of *p*-value < 5e-6 to identify SNPs associated with MPs. Following this step, we implemented a threshold of r^2^ < 0.01 and kb = 1000 to eliminate SNPs in linkage disequilibrium. The resulting SNPs were then harmonized with the GWAS data ukb-b-14486 for OA. After meticulous data harmonization, we obtained a total of 711 SNPs, corresponding to 65 exposures. Remarkably, it is worth noting that all SNPs in this secondary analysis exhibited F statistics exceeding 10, indicating that the majority of the SNPs in this analysis possessed robust instrumental effects. This strategic choice of strong IVs served to minimize potential biases, and further details can be found in Table S6.

**Fig. 3:**
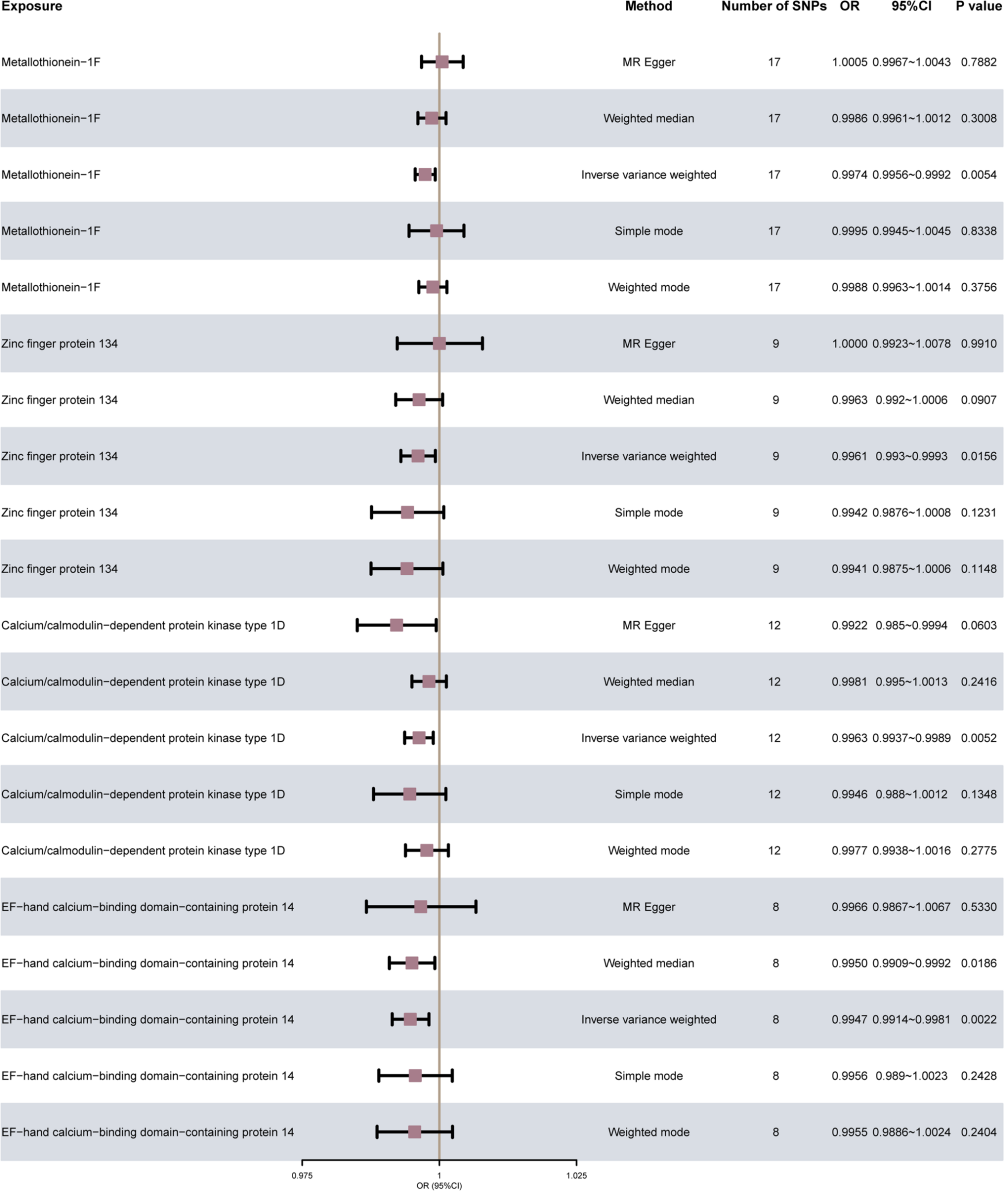
Estimation of causal effects between MPs and OA using different MR methods in second analysis. The forest plot shows the results of multiple Mendelian randomization model (method) analysis of the causal association between MPs (exposure) and osteoarthritis (OA), and the effect evaluation value is performed by OR (95% CI) display, and display the number of SNPs of each model at the same time (number of SNPs) and *p* value (*P* value) value. MR, Mendelian randomization; SNP, single nucleotide polymorphism; OA, osteoarthritis; OR, odds ratio; CI, confidence interval

A total of 5 distinct methodologies, including the IVW approach, MR-Egger, weighted median, simple mode, and weighted mode, were employed to estimate the causal effects and demonstrate the significance of various MPs. The results are shown in Fig. 3. When the number of SNPs ≥ 3, the IVW indicated that 4 MPs, MT-1F, ZNF134, CAMK1D, and EFCAB14 have a significant relationship with the occurrence of OA Causality (*p* value < 0.05), wherein the OR _IVW_ (95% CI) value of MT-1F is 0.9974 (0.9956 ~ 0.9992), the OR _IVW_ (95% CI) value of ZNF134 is 0.9961 (0.993 ~ 0.9993), the OR _IVW_ (95%CI) value of CAMK1D was 0.9963 (0.9937 ~ 0.9989), and the OR _IVW_ (9 5% CI) value of EFCAB14 was 0.9947 (0.9914 ~ 0.9981).

**Fig. 4:**
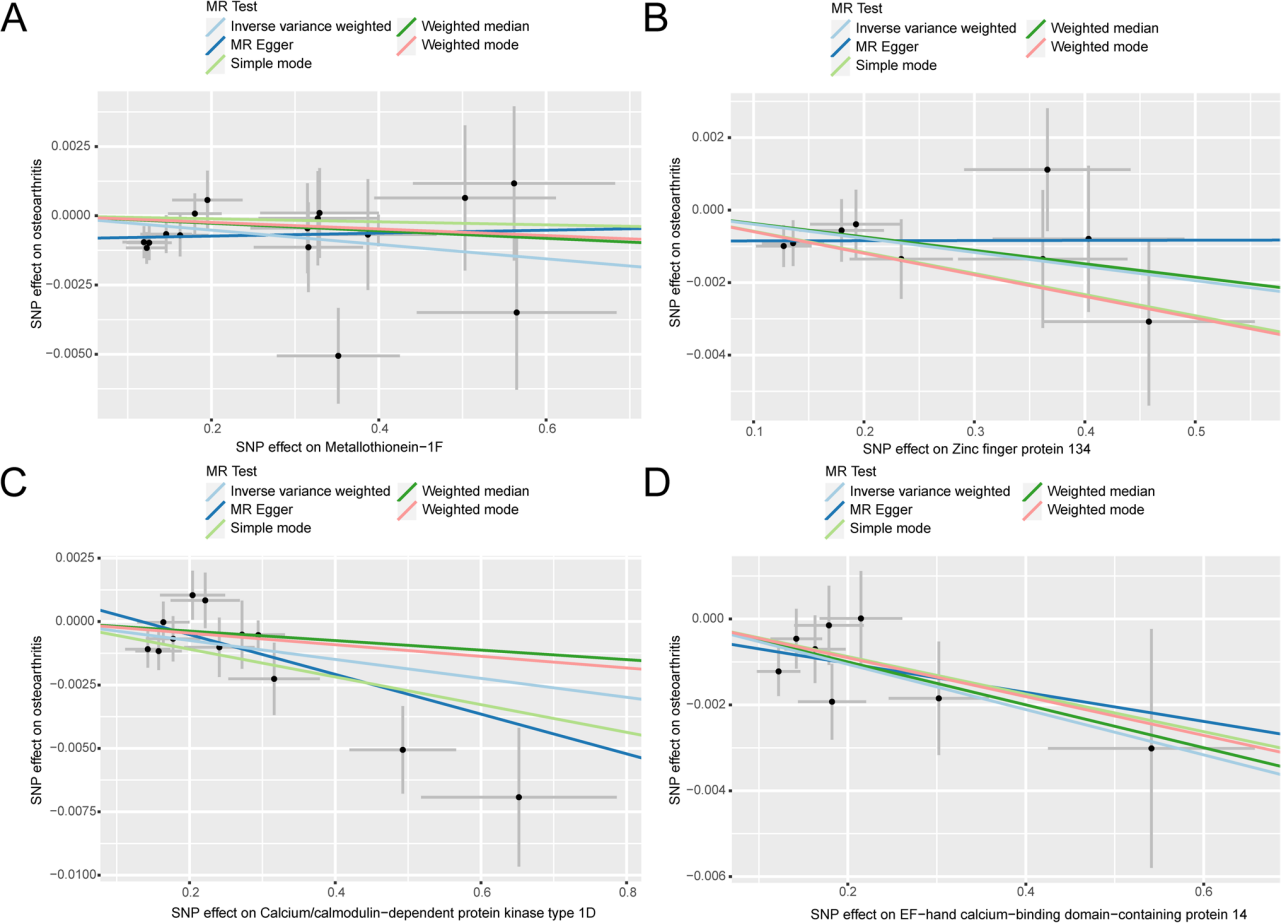
MR results of MPs and OA. **A–D** Five kinds of MR methods were used for metallothionein-1F (**A**), zinc finger protein 134 (**B**), calcium/calmodulin-dependent protein kinase type 1D (**C**), EF-hand calcium-binding domain-containing protein 14 (**D**). Scatter plot of causal effect analysis with OA. OA, osteoarthritis; MPs, metalloproteins; MR, Mendelian randomization

At the same time, we drew a scatter plot (Fig. 4) to show the analysis results of five Mendelian randomization (MR) models; the results showed the MPs MT-1F (Fig. 4A), ZNF 134 (Fig. 4B), CAMK1D (Fig. 4C), and EFCAB14 (Fig. 4D); the estimated slopes of the different MR models were more consistent.

Subsequently, we tested for heterogeneity of significant results using the Cochran Q test and the I^2^ statistic (Table 1). The results show that the Cochran Q test *p*-value of MPs: MT-1F, ZNF 134, CAMK1D, and EFCAB14 > 0.05 and 0 < *I* ^2^ ≤ 0.25, indicating that there is no heterogeneity in the SNPs included in this MR analysis.

**Table 1:** MR heterogeneity test for the association between MPs and OA

Exposure	Method	*Q*	Q df	*P* value	*I*^2^
Metallothionein-1F	MR Egger	12.1509	15	0.6676	0
Metallothionein-1F	Inverse variance weighted	15.5459	16	0.4851	0.0517
Zinc finger protein 134	MR Egger	2.9589	7	0.8888	0
Zinc finger protein 134	Inverse variance weighted	4.1401	8	0.8443	0
Calcium/calmodulin-dependent protein kinase type 1D	MR Egger	13.4702	10	0.1986	0
Calcium/calmodulin-dependent protein kinase type 1D	Inverse variance weighted	15.3634	11	0.1665	0.0517
EF-hand calcium-binding domain-containing protein 14	MR Egger	4.1261	6	0.6596	0
EF-hand calcium-binding domain-containing protein 14	Inverse variance weighted	4.2811	7	0.7469	0

*MR* Mendelian randomization, *OA* osteoarthritis, *Q* Cochran’s Q test statistic, *Q df* degrees of freedom for the Q test, *I*^2^ statistic reflects the proportion of heterogeneity attributed to IVs in the total variability

Subsequently, the MR-Egger intercept test was used to conduct a pleiotropic test (Table 2) on the IVs SNP, and the statistical hypothesis test of the intercept item of each index *p* value > 0.05, the intercept is close to 0, indicating that the causal inference in this study is not affected by horizontal pleiotropy.

**Table 2:** MR horizontal pleiotropy test for the association between MPs and OA

Exposure	MR-Egger intercept	Standard error	*P* value
Metallothionein-1F	-0.0008	0.0005	0.0852
Zinc finger protein 134	-0.0009	0.0008	0.3131
Calcium/calmodulin-dependent protein kinase type 1D	0.0011	0.0009	0.2632
EF-hand calcium-binding domain-containing protein 14	-0.0004	0.0009	0.7074

*OA* osteoarthritis, *MR* Mendelian randomization

**Fig. 5:**
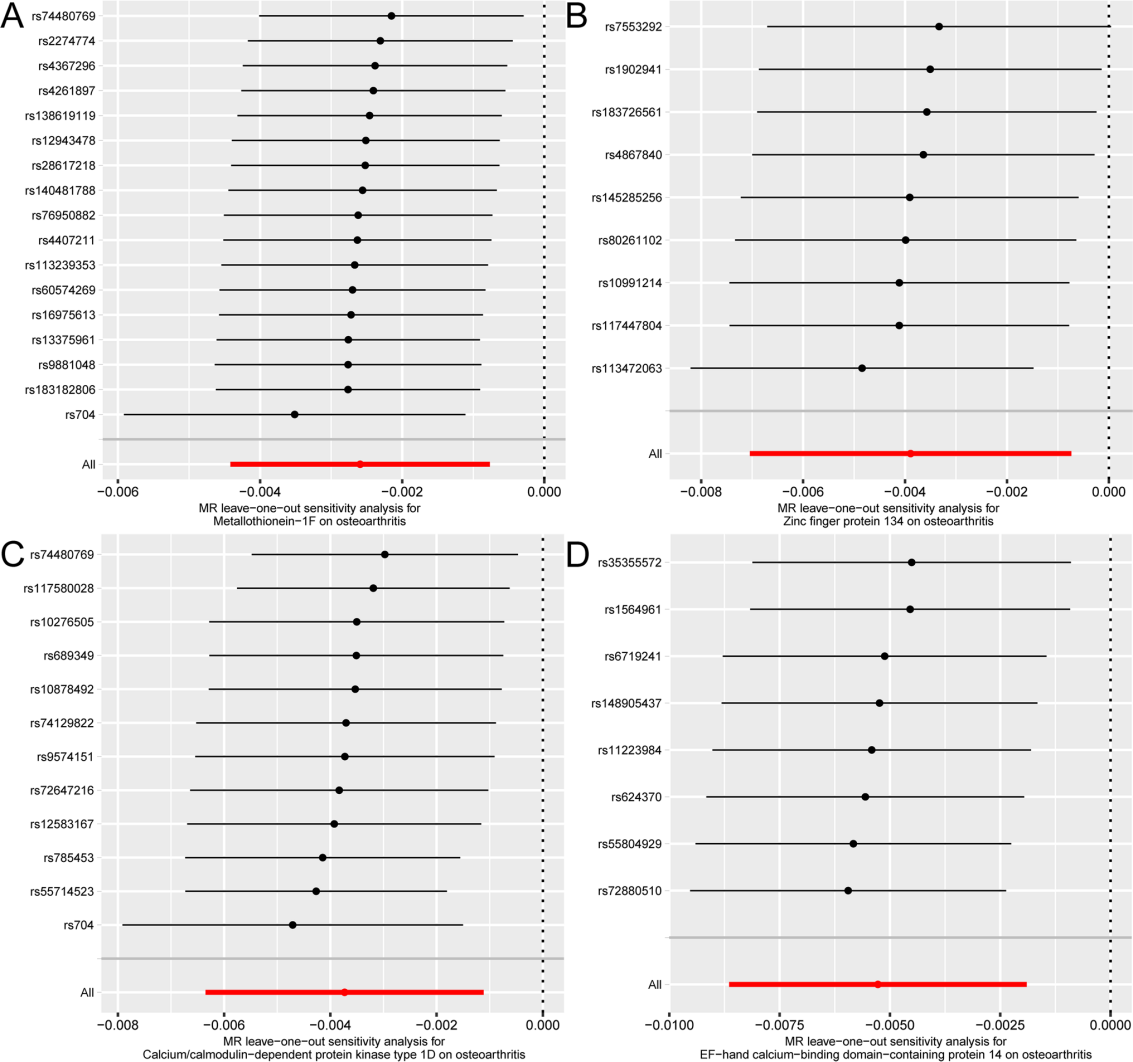
Leave-one-out analysis results. **A–D** Metallothionein -1F (**A**), zinc finger protein 134 (**B**), calcium/calmodulin-dependent protein kinase type 1D (**C**), and EF-hand calcium-binding domain-containing protein 14 (**D**) are eliminated one by one (leave-one-out) forest plot of test analysis results. The vertical axis is the SNP number, and the left side of the horizontal axis is the B (Beta) value. OA, osteoarthritis; MR, Mendelian randomization; SNP, single nucleotide polymorphism

Finally, we performed a sensitivity analysis on the results (Fig. 5), MT-1F (Fig. 5A), ZNF 134 (Fig. 5B), CAMK1D (Fig. 5C), and the total effect of EFCAB14 (Fig. 5D) that has no significant change in B (Beta) value after removing a certain SNP, suggesting that the result is stable.

### Bidirectional Mendelian randomization analysis

**Fig. 6:**
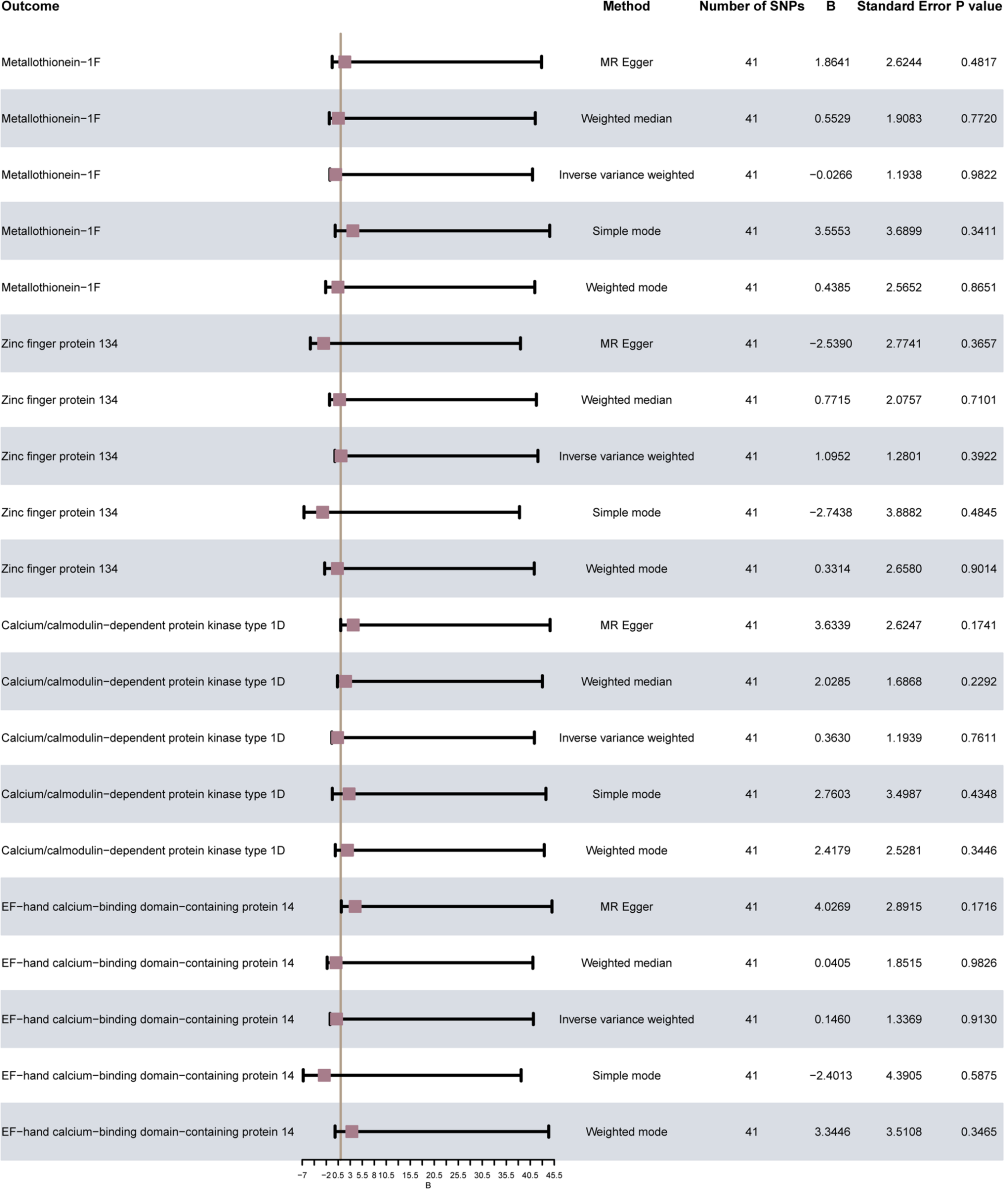
Estimation of causal effects between OA and MPs in bidirectional MR. The forest plot displays the results of various MR model analyses (method) for the causal association between OA and MPs (outcome). The effect evaluation value utilizes B (Beta) value, accompanied by its corresponding standard error (standard error). Additionally, the number of SNPs (number) in each model, as well as the *p* value (*P* value) are presented. OA, osteoarthritis; MPs, metalloproteins; MR, Mendelian randomization; SNP, single nucleotide polymorphism; B, Beta value

To investigate the potential inverse impact of OA on MPs, we designated OA as the exposure factor and assessed its effect on MT-1F, ZNF134, CAMK1D, and EFCAB14 as outcome variables. We employed five distinct MR methods, including IVW, MR-Egger, weighted median, simple mode, and weighted mode, for causal effect estimation analysis. The results are visualized in Fig. 6. The results showed that five MR methods showed that OA had no significant effect on four MPs (*p*-value > 0.05), which further proved the causal effect of the four MPs on OA.

### Multivariate Mendelian randomization

We added four exposure factors (nutritional anemias), semilunar cartilage resection (endoscopic resection of semilunar cartilage NEC), malnutrition (malnutrition), and excessive menstruation (excessive and frequent menstruation with regular cycle), and four MPs, MT-1F, ZNF 134, CAMK1D, and EFCAB14 were subjected to MVMR analysis to assess the direct effects of significant MPs on OA.

The IVs (SNP) of ZNF134 and EFCAB14 in the model were all eliminated and therefore not included in the multivariate analysis. According to the results shown (Table 3). In Model 1, the indirect effect of nutritional anemia (nutritional anemias) was corrected, and MT-1F had a significant effect on OA. In the indirect effect of endoscopic resection of semilunar cartilage NEC (id: ukb-b-7881), CAMK1D still has a significant effect on OA, corrected in Model 3. Despite the indirect effects of malnutrition (malnutrition), MT-1F still has a significant effect on OA; in Model 4, it corrected the effects of excessive and frequent menstruation with regular cycle indirect effect; MT-1F still has a significant effect on OA.

**Table 3:** The results of MVMR analysis of MPs on the incidence of OA

Exposure	Number of SNPs	OR	95%IC	*P* value
Model 1				
Metallothionein-1F	2	0.9988	0.9977 ~ 1.0000	0.0408
Nutritional anemias	1	1.0006	0.9969 ~ 1.0043	0.7484
Model 2				
Calcium/calmodulin-dependent protein kinase type 1D	2	0.9971	0.9947 ~ 0.9995	0.0183
Endoscopic resection of semilunar cartilage NEC	1	3.9887	2.4918 ~ 6.3847	0
Model 3				
Metallothionein-1F	2	0.9988	0.9977 ~ 1.0000	0.0416
Malnutrition	1	1.0002	1.0000 ~ 1.0003	0.0109
Model 4				
Metallothionein-1F	2	0.9989	0.9979 ~ 0.9999	0.0246
Excessive and frequent menstruation with regular cycle	1	0.7393	0.5687 ~ 0.9612	0.0241

*MVMR* multivariable Mendelian randomization, *OA* osteoarthritis

## Discussion

In this pioneering study, we employed a two-sample MR approach to systematically explore the putative causal relationship between MPs and OA. Our analysis highlighted that heightened levels of MT-1F, ZNF134, CAMK1D, and EFCAB14 could be inversely correlated with OA risk. Yet, the association between OA risk and other MPs did not emerge as consistently robust. To examine potential reverse causality—wherein OA could modulate MPs expressions—we applied five distinct MR methods. These consistently revealed no notable influence of OA on the previously mentioned four MPs (*p* > 0.05). Remarkably, a MVMR analysis affirmed the significant impact of MPs on OA, even after accounting for four confounding factors.

Metallothioneins (MTs) stand out as pivotal players in immunosuppressive pathways. Their expression can be triggered by a plethora of biological and environmental stimuli, encompassing heavy metals, oxidative stress, and hypoxia [23]. A defining pathological feature of OA is the degenerative shifts in articular cartilage, marked by either aberrant chondrocyte proliferation leading to cluster formations or a decline in cellular count owing to apoptosis [24]. The upregulation of MTs in OA cartilage has been reported and confirmed in previous studies [8]. Prior research has demonstrated that MT-1 could inhibit the expression of pro-inflammatory cytokines, thereby impeding the progression of OA [9]. Through MR analysis, we have deduced that MT-1F plays a protective role in the occurrence of arthritis. Given that MT-1F is one of the subtypes of MT-1, this suggests that MT-1F continues to exert inhibitory effects on the development of OA.

Zinc finger proteins (ZNFs) represent one of the most abundant protein families known to date, with pivotal roles in numerous biological processes. Their functions are extraordinarily diverse and include DNA recognition, RNA packaging, transcriptional activation, regulation of apoptosis, protein folding and assembly, and lipid binding [25]. Extensive research has unveiled associations between various ZNF subtypes and OA. For instance, ZNF440 has been demonstrated to modulate the expression of inflammatory, catabolic, and apoptotic markers in OA chondrocytes, implying its involvement in the pathogenic mechanisms underlying cartilage degeneration in OA [26]. Conversely, ZNF521 exerts its anti-OA effects by regulating the levels of nuclear histone deacetylase 4 [27]. Additionally, ZNFA20 exhibits reduced expression in OA cartilage samples [28]. However, its overexpression has been shown to ameliorate extracellular matrix degradation and apoptosis in chondrocytes in vitro, as well as reduce inflammation in OA mice. These findings underscore the distinct impacts of different ZNF subtypes on OA. Furthermore, our MR analysis has, for the first time, provided genetic evidence supporting a potential protective role of ZNF134 against OA. This study contributes to a deeper understanding of the role of ZNF134 in the pathogenesis of OA, offering valuable insights for future research and therapeutic approaches.

CAMK1D is ubiquitously present in various cell types, and prior research has highlighted the pivotal functions of CAMK1D in the calcium-triggered CaMKK-CaMK1 signaling cascade [29]. It governs calcium-mediated granulocyte function and respiratory burst [30] while also fostering the basal dendrite growth of hippocampal neurons [31]. However, CAMK1D exhibits varying functions across different disease types. Prior investigations have elucidated CAMK1D’s role in promoting glioma cell proliferation, migration, and invasion through the activation of the PI3K/AKT/mTOR signaling pathway [32]. In recent years, mounting evidence has underscored the regulatory roles of autophagy and apoptosis in the pathogenesis of OA [33]. Chondrocytes have the capacity to modulate autophagy and apoptosis via the PI3K/Akt/mTOR pathway, offering potential avenues for improving OA outcomes [24]. Interestingly, our MR analysis has revealed a suppressive effect of CAMK1D on OA development. However, the research on the association between CAMK1D and OA remains limited and warrants further investigation.

Concurrently, our understanding of EFCAB14 remains limited. According to the GenBank, EFCAB14 (NM014774.3,NP055589.1) is potentially a membrane component with calcium ion-binding activity. It is widely recognized that cell membrane surface proteins serve pivotal functions, encompassing intercellular signal transduction and cellular interaction with the extracellular milieu. EFCAB14 is poised, through a yet-to-be-elucidated mechanism, to potentially attenuate the progression of OA. Nonetheless, rigorous further investigation is imperative to substantiate this proposition. Based on our research findings, we propose, for the first time, a causal relationship between EFCAB14 and OA. This discovery might open up novel avenues for the prevention and treatment of OA.

This study conducted a comprehensive investigation into the causal relationship between MPs and OA using a bidirectional MR design. We utilized genetic tools selected from a substantial OA case dataset and the largest scale of causation related to MPs.

MR analysis is a method employed to reduce potential confounding and reverse causation. It achieves this by considering the random allocation of genotypes from parents to offspring, ultimately producing more robust results. In our study, we employed a variety of methods to assess potential biases stemming from unbalanced horizontal pleiotropy. We also tested the potential associations between IVs and factors that could contribute to specific horizontal pleiotropic pathways. Furthermore, we conducted a MVMR analysis to account for confounding variables. The consistency of our final results provides strong support for our conclusions, highlighting the high precision and stability of our MR analysis.

Nevertheless, this study possesses several limitations. To begin with, the realm of MPs is extensive, yet our analysis focused solely on five specific types. Consequently, the associations between OA and various unexamined MPs remain uncharted and necessitate additional scrutiny and investigation. Secondly, given the relatively sparse availability of high-tier evidence in basic research pertaining to the association between MPs and OA, it is imperative that our present findings undergo additional verification within multicenter cohorts. Furthermore, a comprehensive exploration would benefit from the incorporation of high-throughput sequencing analysis of human samples. Thirdly, our study did not take into consideration the diversity within the spectrum of OA, nor did it conduct an analysis of site-specific OA. Fourthly, as our study’s data originates from European sources, the generalizability of our findings is constrained to European cohorts, and their relevance to other population groups warrants further observation and investigation.

To the best of our knowledge, the current study represents the most extensive genetic correlation investigation between MPs (ZNFs, FRP, CRP, MMPs, MTs) and OA. The study findings reveal a negative causal relationship between MPs (MT-1F, ZNF134, CAMK1D, EFCAB14) and OA. These results provide novel insights into the genetics of OA.

### Supplementary Information

Below is the link to the electronic supplementary material.

Supplementary file1 (CSV 13 KB)

Supplementary file2 (XLSX 9 KB)

Supplementary file3 (CSV 35 KB)

Supplementary file4 (CSV 1 KB)

Supplementary file5 (CSV 1 KB)

Supplementary file6 (CSV 365 KB)

## Data Availability

The data underlying this article are available in the article and in its online supplementary material.
